# Prevalence of pathologies related to impacted mandibular third molars

**DOI:** 10.1186/s40064-016-2640-4

**Published:** 2016-06-29

**Authors:** Seung-Min Shin, Eun Joo Choi, Seong-Yong Moon

**Affiliations:** Department of Oral and Maxillofacial Surgery, School of Dentistry, Chosun University, 375, SeoSukDong, DongGu, GwangJu City, 501-759 Republic of Korea; Department of Oral and Maxillofacial Surgery, School of Dentistry, Wonkwang University, Iksan, Republic of Korea

**Keywords:** Impacted third molar, Pathological lesion, Dentigerous cyst, Ameloblastoma, Surgical extraction

## Abstract

**Objective:**

Prevalence of cysts and tumors related to impacted third molars has been considered important because the risk justifies prophylactic extraction. This retrospective study aimed to evaluate the prevalence of cysts or tumors associated with impacted mandibular third molars (IMTMs) according to patients’ age and gender.

**Methods:**

Over the period from August 2006 to August 2011, 20,802 third molars from 17,535 patients were removed. Among these, IMTMs without cysts nor tumors were classified as non-pathology group, and IMTMs with cysts and tumors were classified into pathology group. The prevalence of IMTMs and associated cysts or tumors was analyzed in patient groups stratified by age and gender. The pathology group patients were also classified according to histopathological findings and the corresponding age groups.

**Results:**

Radiographic signs of disease were detected for 176 lesions (0.846 %) in 165 patients. Of these, 135 (76.4 %) lesions were diagnosed as dentigerous cysts, 31 (17.6 %) as keratocysticodontogenic tumors, and 10 (5.7 %) as ameloblastomas. The prevalence of cysts or tumors tended to increase after 50 years of age, such as 7.27 % in 6th decades, 18.60 % in 7th decades, and 11.53 % in 8th decades, with a male predominance in older patients.

**Conclusions:**

IMTMs in old age patients more than 50 years old has high possibilities of developing cyst or tumors especially in male patients. However, these results should not be used as the only evidence for justifying prophylactic extraction, and further studies should investigate the survival rate of IMTMs without any pathologic in older populations.

## Background

Although recent studies have investigated the benefits of prophylactic extraction of impacted mandibular third molars (IMTMs), the indications for this procedure remain controversial (Steed 2014) because surgical extraction can result in numerous complications, including nerve damage, infection, and impaired healing in older patients (Baykul et al. 2005). Indications for therapeutic extraction of impacted third molars include recurrent pericoronitis, cysts, nonrestorable caries lesions, and destruction of adjacent periodontal tissues (National Institute for Health and Care Excellence 2000).

Previous literatures reported that cysts and tumors develop around IMTMs in fact with low incidence. The incidence of cysts and tumors ranges from 2 to 6.2 % in most studies (Goldberg et al. 1985; Stathopoulos et al. 2011; Lysell and Rohlin 1988; Samsudin and Mason 1994; Nordenram et al. 1987; Bruce et al. 1980). There are rare reports that high incidence of cyst or tumor formation in the oldest age group with mean age of 46.5 years old (13.3 %) rather than youngest age group with mean age of 20 years (1.5 %).

However, it has not been investigated that incidence of cyst or tumor is changed according to the patients’ age and gender. This retrospective study aims to assess the prevalence of cysts or tumors adjacent to IMTMs according to age and gender.

## Subjects and methods

Over the period from August 2006 to August 2011, 20,802 third molars from 17,535 patients were removed at the Department of Oral and Maxillofacial Surgery. The decision on extraction was made by patients after being informed of surgical risks such as nerve damage, infection, and delayed healing. The patients were also informed about the potential risk of unextracted IMTMs. Removed all IMTMs were included in this study, and IMTMs without any pathologic lesion, radiographically a radiolucency less than 3 mm were classified into Group A (Table 1), and IMTMs with pathologies, radiographically a radiolucency more than 3 mm were classified into Group B. All of the Group A patients wanted to extract by their willing and impaction in the bone of IMTM was included and excluded only covered with soft tissue in this study.

**Table 1:** Incidence of cysts or tumors adjacent to impacted mandibular third molars according to age and gender

Age (years)	Group A			Group B			Total
	Male	Female	Total	Male	Female	Total	
10–19	1721	1635	3356	16	8	24 (0.71 %)^a^	3380 (16.248 %)^b^
20–29	7019	5628	12,647	34	19	53 (0.41 %)^a^	12,700 (61.051 %)^b^
30–39	1950	1258	3208	26	8	34 (1.04 %)^a^	3242 (15.585 %)^b^
40–49	747	282	1029	18	5	23 (2.18 %)^a^	1052 (5.057 %)^b^
50–59	200	93	293	20	3	23 (7.27 %)^a^	316 (1.519 %)^b^
60–69	54	16	70	14	2	16 (18.60 %)^a^	86 (0.414 %)^b^
70–	13	10	23	3	0	3 (11.53 %)^a^	26 (0.125 %)^b^
Total	11,704	8922	20,626	131	45	176	20,802

Group A: patients who underwent surgical extraction of IMTMs

Group B: patients diagnosed with cysts or tumors adjacent to IMTMs who underwent surgical removal

^a^The percentage is calculated to divide the patients in Group B into total patients for each age group

^b^The percentage is calculated to divide the number of patients in each age group into the total number of patients

Panoramic and computed tomography were performed to diagnose impaction status, and to judge the proximity of the IMTMs to the cysts or tumors. All patients’ medical records were reviewed for information such as age and gender. The histopathological reports on the lesion related to Groups A, B IMTMs were also reviewed.

The patients were classified according to their age and gender. The prevalence of IMTMs was analyzed for each age group and compared with the overall incidence. For each age of Group B, the prevalence of cysts or tumors was analyzed and compared with the overall prevalence in patients with IMTMs. Male and female predilection was analyzed for each age group, and the Group B patients were classified according to the histopathological results and the corresponding age groups.

This protocol was approved by the Chosun University Dental Hospital Ethics Committee (CDMDIRB-1322-112).

## Results

A total of 20,802 third molars from 17,535 patients were included in this study. 165 patients had 176 pathologic IMTMs, including 11 bilateral lesions associated with bilateral IMTMs.

### General patient characteristics

There were 11,970 male patients and 9008 female patients. The overall patient age ranged from 13 to 88 years, with a mean of 26.1 years. The rate of extraction was 61.1 % for patients aged 20–29 years, 16.2 % for those aged 10–19 years, and 15.6 % for those aged 30–39 years (Table 1).

### Characteristics of Group B patients

On histopathological analysis, 176 pathological lesions (0.846 %) in 165 patients were identified as a cyst and tumor associated with an IMTM. The mean age of these patients was 37.2 years, with an age range of 13–78 years. Patients aged 20–29 years were the most frequently affected, followed by patients aged 30–39 years.

### Prevalence of cysts or tumors according to age

The number of patients diagnosed with cysts or tumors was lower among those aged >30 years than among those aged ≤30 years. However, the proportion of cysts or tumors associated with extracted IMTMs was found to increase with age; it was 0.41–0.71 % among patients aged ≤30 years and 7.69–20.93 % among patients aged >50 years.

### Prevalence of cysts or tumors according to gender

The prevalence of cysts or tumors was considerably higher among the male patients than among the female patients (male:female, 3.29:1, Table 2), although it should be considered that the number of male patients that underwent extraction of IMTMs was higher than that of female patients (1.32:1). Table 2 shows that the prevalence of cysts and tumors considerably increased with age in male patients, but not in female patients.

**Table 2:** Comparison of the incidence ratio for male and female patients in Groups A and B

Male:female (Group A)	Age (years)	Male:female (Group B)
1.06:1	10–19	1.75:1
1.25:1	20–29	1.53:1
1.56:1	30–39	4:1
2.67:1	40–49	4.5:1
2.29:1	50–59	28:1
3.78:1	60–69	8:1
1.6:1	70–	∞^a^
1.32:1	Total	3.29:1

Group A: patients who underwent surgical extraction of impacted mandibular third molars with no pathologies

Group B: patients diagnosed with cysts and tumors adjacent to impacted mandibular third molars who underwent surgical removal

^a^The incidence ratio is not calculated because no female patient over the age of 70 was diagnosed with cysts or tumors adjacent to impacted mandibular third molars

**Fig. 1:**
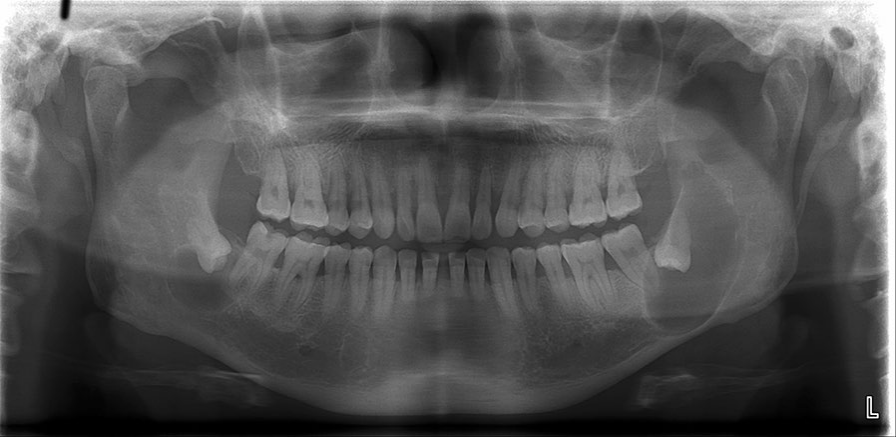
Well-defined pericoronal radiolucencies observed on radiographs of teeth #38 and #48 in a 50-year-old man. The lesions were later diagnosed as dentigerous cysts by histopathologic examination

**Fig. 2:**
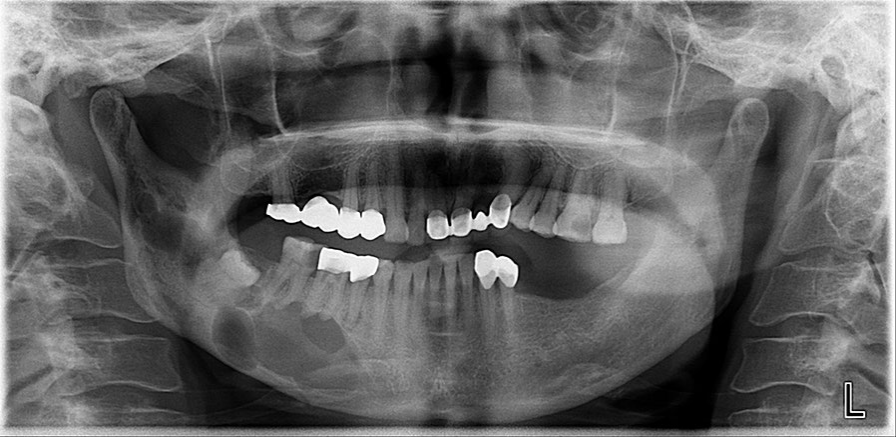
Multilocular radiolucency extending from tooth #45 to the right ascending ramus, with impaction of tooth #48 in a 59-year-old woman. The lesion was diagnosed as a keratocysticodontogenic tumor by histopathologic examination

**Fig. 3:**
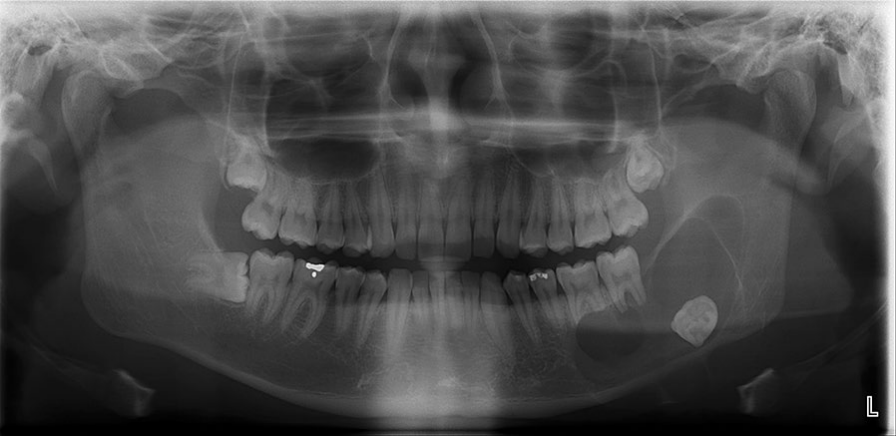
Well-defined radiolucency extending from tooth #36 to the left ascending ramus, with impaction of tooth #38 in a 19-year-old-man. The lesion was diagnosed as an ameloblastoma by histopathologic examination

Of the 176 lesions, 135 (76.4 %) were diagnosed as dentigerous cysts, 31 (17.6 %) as keratocystic odontogenic tumors, and 10 (5.7 %) as ameloblastomas. Eleven of these lesions were bilateral (Table 3; Figs. 1, 2, 3).

**Table 3:** Histopathological findings of cysts or tumors adjacent to impacted mandibular third molars

Age	Dentigerous cyst	Kerato-cystic odontogenic tumor	Ameloblastoma	Total
10–19	12	9	1	22
20–29	38	7	3	48
30–39	30	4	1	35
40–49	16	5	1	22
50–59	25	4	0	29
60–69	13	1	4	18
70–	1	1	0	2
Total	135 (76.7 %)	31 (17.6 %)	10 (5.7 %)	176 (100 %)

## Discussion

The indications for prophylactic extraction of asymptomatic impacted third molars remain controversial because of the potential for complications such as postoperative swelling, trismus, fracture, and nerve injury (Steed 2014; Baykul et al. 2005). The indications for therapeutic extraction of impacted third molars include recurrent pericoronitis, cellulitis, abscess, osteomyelitis, follicular diseases such as cysts and tumors, nonrestorable caries, irreversible pulp or periapical pathology, internal or external resorption of adjacent teeth, tooth fracture, interference with impending surgery or reconstructive jaw surgery, and tumor resection (National Institute for Health and Care Excellence 2000).

Among these pathological conditions, the prevalence of cysts or tumors associated with impacted third molars reportedly ranges from 2 to 6.2 % (Table 4; Goldberg et al. 1985; Lysell and Rohlin 1988; Samsudin and Mason 1994; Nordenram et al. 1987; Bruce et al. 1980; Al-Khateeb and Bataineh 2006; Brickley et al. 1995; Güven et al. 2000). The prevalence in the present study was 0.846 %, which is considerably lower than that reported previously. In addition, the number of patients diagnosed with cysts or tumors was lower in patients aged >30 years. However, the proportion of cyst or tumors associated with IMTMs increased with age. These findings indicate that older patients are at a higher risk of developing cysts or tumors associated with IMTMs.

**Table 4:** Reported prevalence of cysts or tumors associated with impacted mandibular third molars

References	Incidence (%)
Bruce et al. (1980)	6.2
Goldberg et al. (1985)	2
Nordenram et al. (1987)	4.5
Lysell and Rohlin (1988)	3
Samsudin and Mason (1994)	3.3
Brickley et al. (1995)	3.5
Güven et al. (2000)	3.1
Al-Khateeb and Bataineh (2006)	2.6
Current study (2016)	0.864

IMTMs are frequently extracted from prophylactic reasons in patients aged ≤30 years and rarely extracted in patients aged >40 years because of the high complication rate (Osborn et al. 1985). Therefore, we believe that there were more cases of prophylactic extraction in younger patients than in older patients in Group A. This possible explained the lower proportion (0.41–0.71 %) of cysts or tumors in patient aged ≤30 years. However, the prevalence of cysts or tumors tended to increase after the age of 50 years with 7.27 % in 6th decades, 18.60 % in 7th decades, and 11.53 % in 8th decades; this finding was in accordance with that of a previous study, where a cyst was the indication for extraction in a fourth of the patients aged >50 years (Knutsson et al. 1996).

Because Group A includes only patients who underwent extraction of IMTMs, not patients simply diagnosed with IMTMs, the prevalence of cysts or tumors was not compared with that in all patients with IMTMs. However, the design of this study was such that the prevalence of cysts or tumors could be overestimated because patients with IMTMs and no other pathology or symptoms were excluded. There may have been several pathologies in the Group A patients, including recurrent pericoronitis, cellulitis, abscess, osteomyelitis, nonrestorable caries, irreversible pulp or periapical pathology, internal or external resorption of adjacent teeth, and tooth fracture (National Institute for Health and Care Excellence 2000). Therefore, these results cannot be used as the only evidence justifying prophylactic extraction in young patients, and further studies should investigate the survival rate of IMTMs without any complications to older patients.

Several theories have been suggested to explain the development of odontogenic cysts such as dentigerous cysts and keratocysticodontogenic tumors. One suggests that long-standing inflammation results of cystic lesions (Lin et al. 2013), while another suggests the role of mutations of specific genes (Cabay 2014). According to these theories, the higher prevalence of cysts or tumors in older age groups is probably a result of sustained long-standing inflammatory processes or the possible accumulation of genetic mutations.

We found that the male patients exhibited a considerably higher prevalence of cysts or tumors compared with the female patients (3.29:1), although the number of male patients who underwent extraction of IMTMs was considerably higher than that of female patients (1.32:1). Table 2 shows that the prevalence of cysts and tumors considerably increased with age in male patients, but not in female patients. The reason for gender predilections, particularly among older patients, is not clear. Because IMTMs are generally removed from prophylactic purposes in young patients (10–29 years) in the Republic of Korea, the proportion of retained IMTMs is lower in older age groups. The difference in prevalence according to age between the male and female patients could be biased by a small number of patients in the older age groups. In addition, dentigerous cysts develop with a male predominance in Asian populations (Lin et al. 2013; Selvamani et al. 2012). Although the reasons for the male predilection of cysts or tumors remains unclear, increased consumption of alcohol or tobacco among male patients in Asia is believed to affect genetic mutations related to the development of cysts or tumors.

In this study, specimens from patients who were not radiologically diagnosed with cysts or tumors were not sent for histopathology. Several studies have reported that the histological examination of all follicular tissue after IMTM extraction can reveal dentigerous cysts in patients without radiological evidence of cystic changes (Brickley et al. 1995; Adelsperger et al. 2000; Zhang et al. 2010); the prevalence of cysts or tumors in the present study could thus be relatively low. If all patients in this study had undergone histopathological examination, the prevalence rate may have been higher.

Dentigerous cysts frequently develop adjacent to IMTMs and can progress to more serious lesions such as keratocysticodontogenic tumors or unicystic ameloblastomas (Zhang et al. 2010). In the present study, 135 (76.4 %) lesions were diagnosed as dentigerous cysts, 31(17.6 %) as keratocysticodontogenic tumors, and 10 (5.7 %) as ameloblastomas; nine dentigerous cysts and two keratocysticodontogenic tumors were bilateral.

## Conclusions

IMTMs in old aged patients more than 50 years old has high possibilities of developing cyst or tumors especially in male patients. However, these results should not be used as the only evidence for justifying prophylactic extraction, and further studies should investigate the survival rate of IMTMs without any pathology in older populations.
